# ASSOCIATION OF HEALTH BEHAVIORS WITH WEIGHT AND OBESITY DURING THE COVID-19 PANDEMIC AMONG THE UK OLDER POPULATION

**DOI:** 10.1093/geroni/igac059.763

**Published:** 2022-12-20

**Authors:** Jingmin Zhu, Giorgio Di Gessa, Paola Zaninotto

**Affiliations:** UCL, London, England, United Kingdom; UCL, London, England, United Kingdom; UCL, London, England, United Kingdom

## Abstract

Using a sample of 4,182 UK adults aged 50 and above, this study explored the association of changes in health behaviours with weight and obesity during UK lockdown in Jun/Jul and Nov/Dec 2020. Over 30% adults reported more sitting, more TV watching or less exercise. Around 20% adults were engaged in eating more or sleeping less. More alcohol drinking happened in 12.3% adults. Results suggested that more sedentariness, more TV watching, less exercise, more eating and more alcohol drinking were associated with a significant increase in weight. Meanwhile, less sedentariness or less eating significantly reduced weight in Nov/Dec 2020. A higher risk of obesity was found in adults sitting, eating, or sleeping more than usual. Considering potential health risks associated with obesity in older population, weight management is necessary nationwide.

